# A Multifunctional Region of the *Shigella* Type 3 Effector IpgB1 Is Important for Secretion from Bacteria and Membrane Targeting in Eukaryotic Cells

**DOI:** 10.1371/journal.pone.0093461

**Published:** 2014-04-09

**Authors:** Sonia C. P. Costa, Cammie F. Lesser

**Affiliations:** Department of Medicine (Microbiology and Immunobiology), Division of Infectious Diseases, Massachusetts General Hospital and Harvard Medical School, Cambridge, Massachusetts, United States of America; University of Arkansas for Medical Sciences, United States of America

## Abstract

Type 3 secretion systems are complex nanomachines used by many Gram*–*negative bacteria to deliver tens of proteins (effectors) directly into host cells. Once delivered into host cells, effectors often target to specific cellular loci where they usurp host cell processes to their advantage. Here, using the yeast model system, we identify the membrane localization domain (MLD) of IpgB1, a stretch of 20 amino acids enriched for hydrophobic residues essential for the targeting of this effector to the plasma membrane. Embedded within these residues are ten that define the IpgB1 chaperone-binding domain for Spa15. As observed with dedicated class IA chaperones that mask hydrophobic MLDs, Spa15, a class IB chaperone, promotes IpgB1 stability by binding this hydrophobic region. However, despite being stable, an IpgB1 allele that lacks the MLD is not recognized as a secreted substrate. Similarly, deletion of the chaperone binding domains of IpgB1 and three additional Spa15-dependent effectors result in alleles that are no longer recognized as secreted substrates despite the presence of intact N-terminal secretion signal sequences. This is in contrast with MLD-containing effectors that bind class IA dedicated chaperones, as deletion of the MLD of these effectors alleviates the chaperone requirement for secretion. These observations indicate that at least for substrates of class IB chaperones, the chaperone-effector complex plays a major role in defining type 3 secreted proteins and highlight how a single region of an effector can play important roles both within prokaryotic and eukaryotic cells.

## Introduction

Modulation of host signaling pathways by bacterial pathogens is critical as it enables these microorganisms to colonize and replicate within or in the vicinity of eukaryotic host cells. To facilitate this goal, bacteria have evolved a variety of mechanisms to deliver proteins directly into host cells, proteins that act to usurp host cell processes to promote bacterial survival and spread. For example, over the course of an infection, *Shigella* species, the causative agents of bacillary dysentery, utilize a type 3 secretion system (T3SS) to directly inject at least 30 proteins, referred to as effectors, into intestinal epithelial cells (for review see [1]).

A key step in *Shigella* pathogenesis is the ability of this intracellular pathogen to mediate its own uptake of into normally non-phagocytic epithelial cells. Several type 3 secreted effectors including IpgB1 are involved in this process. IpgB1 is a GEF (GTP exchange factor) [2] that localizes to host cell membranes at bacterial entry sites where it activates the small GTPases Rac and Cdc42 to induce membrane ruffling which promote the uptake of *Shigella* into host cells [3]–[6]. *Shigella* strains that no longer encode IpgB1 are impaired in invasion, produce smaller ruffles and are attenuated in virulence [6].

The translocation (delivery) of IpgB1into host cells is dependent on Spa15, a class IB type 3 secretion chaperone [7]. In addition to IpgB1, Spa15 binds to and mediates the secretion of eight additional *Shigella* effectors [7]–[10]. Interestingly, these nine effectors share a conserved amino acid sequence, the conserved chaperone binding domain (CCBD) sequence, within their first 50 residues that mediates interactions with Spa15 [11].

In this study, using the yeast *Saccharomyces cerevisiae* as a model system [12], [13], we have identified a twenty amino acid region of IpgB1 responsible for its membrane localization. This region, like that of the previously mapped membrane localization domains (MLDs) of several other type 3 effectors, is enriched in hydrophobic residues that likely promote membrane targeting. Included within these twenty residues are ten that define the IpgB1 chaperone binding domain (CBD) [11]. Interestingly, although IpgB1 is one of nine effectors that bind Spa15, the stability of only IpgB1 is dependent on this class IB chaperone. In the absence of Spa15, IpgB1 stability is restored by the deletion of the twenty residues that define its MLD. Thus, as previously observed with other effectors that target host cell membranes, chaperones can act within bacteria to mask hydrophobic MLDs. However, in contrast to other MLD-containing effectors, which are secreted in the absence of their cognate chaperones when their MLDs are deleted, IpgB1 alleles that lack its MLD or just its CBD are no longer secreted. Similarly, at least three additional Spa15-dependent effectors are also no longer recognized as secreted substrates when their CBDs are deleted, despite the presence of an intact N-terminal secretion signal sequence. Thus, our studies demonstrate how a single region of an effector, in this case IpgB1, can play important roles both in defining the protein as a bacterial secreted substrate as well as in promoting membrane localization once delivered into mammalian cells.

## Experimental Procedures

### Plasmids and strains

All of the plasmids encoding wild type alleles of the *Shigella* effectors have been previously described [10], [11], [14]. The mutant and chimeric effector alleles were generated via overlap PCR and the Gateway site-specific recombination system (Invitrogen). Amplified mutant genes flank by attB sites and an upstream Shine-Dalgarno sequence were introduced into pDNR221 via BP reactions (Invitrogen). Each gene introduced into an entry vector was sequence verified and subsequently transferred via an LR reaction (Invitrogen) into a destination expression vector. For expression in yeast, the genes were introduced into pDYST-GFP-ccdB, a high copy number (2μ) plasmid that carries the *LEU2* gene and the *GAL10* promoter [15]. For the bacterial expression studies, genes encoding the specified alleles were introduced into pDSW206-ccdB-FLAG, a low copy (ColE1 ori) ampicillin resistance plasmid that carries an impaired pTac promoter [10]. All the *Shigella* studies were conducted in *S. flexneri* 2457T serotype 2a [10]. The *spa15* and *ipgB1 Shigella* deletion strains were generated using the λred recombination system [16]. All oligonucleotide used in this study are described in Table S1.

### Yeast fluorescence microscopy

Yeast expression plasmids were transformed into S288C using the PEG/lithium acetate method. Yeast carrying the plasmids were grown overnight in SC -Leu media with 2% raffinose as a carbon source. In the morning the cultures were back diluted to an OD_600_ of 0.5 and incubated for an additional 2 hours (h) at which point galactose was added (final concentration of 2%). Four hours post–induction, yeast cells were visualized using a Nikon TE3000 microscope with Chroma Technology filters and a 100x objective. To visualize nuclei, yeast cells were harvested, fixed for 30 minutes (min) with paraformaldehyde, permeabilized with 70% ethanol for 20 min and stained with DAPI for10 min. Images were captured digitally using a black-and-white Sensys charge-coupled-device (CCD) camera and IPLAB software (Scanalytics). Color images were assembled by separately capturing signals with each of the appropriate filter sets and digitally pseudocoloring the images. The bars in the images represent ∼2 μm, half the length of a haploid yeast.

### Bacterial protein preparations

Wild type and *Δspa15 Shigella* strains carrying the designated pDSW206-effector-FLAG plasmids were grown overnight at 37°C in TCS (trypticase soy) broth. Cultures were back-diluted 1∶100 and grown at 37°C, to allow for expression and assembly of an intact type 3 secretion system. When at an OD_600_ = 0.6, expression of the FLAG-tagged effectors was induced by IPTG (100μM) for 30 min. Equivalent numbers of bacteria, based on OD_600_, were harvested and lysed in SDS-PAGE buffer. Protein content was analyzed by immunoblotting with anti-Flag (Sigma) and anti-IpaB antibodies.

### Secretion assays

Secretion assays were carried as previously described in Schmitz et. al.[10]. Briefly, overnight cultures of wild type and *Δspa15 Shigella* carrying the designated pDSW206-effector-FLAG plasmids were back-diluted 1∶100 and incubated on a roller at 37°C until OD_600_ = 0.6. IPTG was added to a final concentration of 100 μM to induce expression of proteins for 30 min. Type 3 secretion was then activated by the Congo red dye for additional 30 min incubation. The supernatant fraction was separated by two centrifugations at 20,000 *g* for 2 min and the proteins in the supernatant of the second centrifugation were precipitated with TCA (10%). Protein content of the supernatant was analyzed by immunoblotting as described above.

## Results

### The N-terminal 50 Residues of IpgB1 are Sufficient to Target Proteins to the Yeast Plasma Membrane

**Figure 1:**
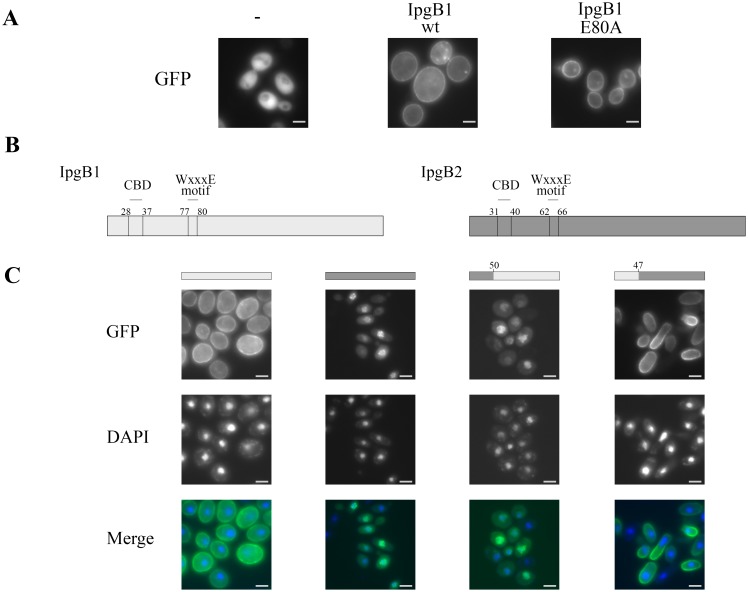
The N-terminal region of IpgB1 is sufficient to target proteins to the yeast plasma membrane. (A, C) Yeast that conditionally express each of the designated fusion proteins under the control of a *GAL1* promoter were visualized by fluorescence microscopy 4 h post-induction of protein expression. The (-) in panel (A) represents free GFP. (B) Schematic representation of IpgB1 and IpgB2. (C) Yeast were fixed and stained with DAPI to visualize nuclear DNA. The images shown are representative of at least 3 independent experiments. All of the GFP positive yeast cells visualized for each condition exhibited the subcellular localization pattern shown.

The yeast *Saccharomyces cerevisiae* is an established model system for studying bacterial virulence proteins, including type 3 secreted effectors. Many effectors exhibit conserved subcellular localization patterns as well as activities when expressed in yeast and mammalian cells [12], [17], [18]. Interestingly, as shown in Figure 1A and in earlier published studies from our group [10], [11], IpgB1 is the only one of over 20 *Shigella* effectors tested that exhibits a membrane subcellular localization pattern when expressed in yeast. This localization reflects conserved subcellular targeting, as IpgB1 localizes to the plasma membrane during the course of an infection or when transiently expressed de novo in mammalian cells [6]. Thus, given that the mechanism that mediates IpgB1 membrane localization is presumably conserved from yeast to humans, we chose to examine this process using the genetically tractable yeast model system.

First, given that IpgB1 is a GEF for Rac and Cdc42 [2] and that many yeast and mammalian GEFs and the GTPases that they bind often localize to membranes, we investigated whether IpgB1 membrane targeting is dependent on its GEF activity. To test this possibility, we compared the yeast subcellular localization patterns of GFP-IpgB1 and GFP-IpgB1_E80A, an allele that carries a mutation in the conserved WxxxE motif. This mutation eliminates GEF activity and based on the solved structures of close IpgB1 homologs is predicted to disrupt the GEF domain [2], [19]. As shown in Figure 1A, wild type IpgB1 and IpgB_E80A both localize to the plasma membrane suggesting that IpgB1 GEF activity does not play a major role in mediating IpgB1 membrane targeting.

IpgB1 promotes *Shigella* uptake and membrane ruffling by recruiting the ELMO-Dock180 complex to membranes [5]. Interestingly, fusion of the first 105 residues of IpgB1 to ELMO is sufficient to generate a protein that localizes to the plasma membrane and promotes the formation of membrane ruffles [5]. To test whether this region of IpgB1 encodes a membrane localization domain, we investigated whether fusion of the first 100 residues of IpgB1 to GFP is sufficient to redirect GFP to the yeast plasma membrane. However, the fusion protein was unstable when expressed in yeast and no fluorescent protein was visible (data not shown). To circumvent this instability issue, we investigated and developed a complementary reciprocal swap strategy to identify the region(s) of IpgB1 sufficient to confer membrane localization to its close structural homolog *Shigella* IpgB2.

IpgB2, like IpgB1, is a Rho GEF and a member of the WxxxE effector family. The predicted secondary structures of IpgB1 and IpgB2 are essentially identical across their GEF domains [4]. In addition, within their first ∼50 amino acids, residues upstream of their respective GEF domains, each effector encodes a variant of the conserved chaperone binding domain sequence, a region that mediates the binding of each to their shared cognate *Shigella* chaperone, Spa15 [11] (Figure 1B). While IpgB1 activates Rac and Cdc42, IpgB2 is a GEF for RhoA [19]. Differences in specificity likely explain why expression of IpgB1 mildly impairs yeast growth, while expression of IpgB2 is highly toxic [14]. Due to this toxicity, wild type alleles of IpgB2 fused to GFP are poorly visualized in yeast [10]. Thus, for the reciprocal swap experiments, we used a catalytically dead allele of IpgB2 (IpgB2_W62A). This allele is mutated at the conserved tryptophan of the WxxxE motif [4] results in loss of GEF activity and completely alleviates yeast toxicity [10]. GFP-IpgB2_W62A, hereafter referred as IpgB2, exhibits a nuclear subcellular localization pattern when expressed in yeast (Figure 1C).

Given that IpgB1 localizes to the plasma membrane independently of its GEF activity, we next investigated whether the N-terminal region of IpgB1 is sufficient to target heterologous proteins to the plasma membrane. Thus, we swapped the regions of IpgB1 and IpgB2 predicted to reside upstream of the first α-helix of their GEF domains, position 47 of IpgB1 and position 50 of IpgB2 [4]. Strikingly, as shown in Figure 1C, the N-terminal reciprocal swaps resulted in a complete switch in the subcellular localization patterns of the two chimeric proteins. IpgB1 with the N-terminal residues of IpgB2 localized to the nucleus, while IpgB2 with the N-terminal residues of IpgB1 localized to the plasma membrane. Therefore, a region encoded within the first 47 residues of IpgB1 plays a major role in directing the localization of IpgB1 as well as heterologous proteins to the plasma membrane.

### Residues within the Chaperone Binding Domains of IpgB1 and IpgB2 Play Major Roles in Determining their Eukaryotic Subcellular Localization Patterns

**Figure 2:**
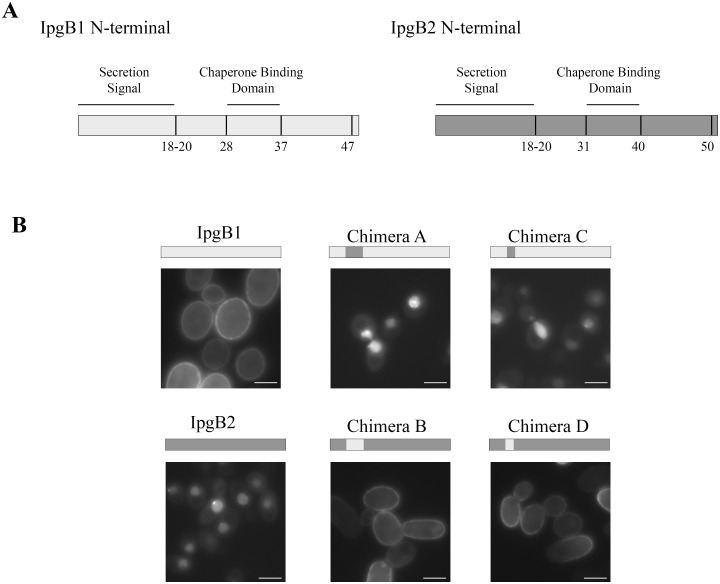
The CBD sequences of IpgB1 and IpgB2 determine their subcellular localization patterns in yeast cells. (A) Schematic representation of the domains located within the N-terminal regions of IpgB1 and IpgB2. (B) Yeast expressing the designated IpgB1 and IpgB2 chimeras were visualized by fluorescence microscopy 4 h post-induction of protein expression. The images shown are representative of at least 3 independent experiments. All of the GFP positive yeast cells visualized for each condition exhibited the subcellular localization pattern shown.

As outlined in Figure 2A, the N-terminal regions that define the unique subcellular localization patterns of IpgB1 and IpgB2 encompass each their type 3 secretion signal and chaperone binding domains sequences [11]. To test whether residues present to either of these domains of IpgB1 are sufficient to direct membrane targeting, we generated smaller reciprocal swaps within the N-terminal residues of the two effectors. First we exchanged a 20 amino acid region that includes the chaperone binding domain (CBD) of each effector plus its downstream 10 amino acids, the residues present before the start of the predicted GEF domain of each. As shown in Fig. 2B (chimeras A and B), the replacement of the 20 residues of one effector with those from the other resulted in a complete switch in their subcellular localization patterns, i.e., the introduction of twenty residues of IpgB1 into IpgB2 resulted in an allele of IpgB2 that localizes to the plasma membrane.

We then generated smaller reciprocal swaps to investigate whether the introduction of the ten residues that define the CBD of IpgB1 is sufficient to direct membrane targeting when introduced into IpgB2 (chimeras C and D). As shown in Figure 2B, the presence of just ten residues of IpgB1, specifically the amino acids that define its CBD [11], is sufficient to generate an allele of IpgB2 that localizes to the plasma membrane. Similarly, the introduction of the ten residues that map to the IpgB2 CBD into IpgB1 resulted in an allele of IpgB1 that localizes to the nucleus (Figure 2B). Thus, while the conserved CBD sequences of IpgB1 and IpgB2 perform identical functions within *Shigella*, the same residues dictate distinct subcellular localizations once the proteins are introduced into eukaryotic cells.

### The CBD of IpgB1 is Located within a Membrane Localization Domain

**Figure 3:**
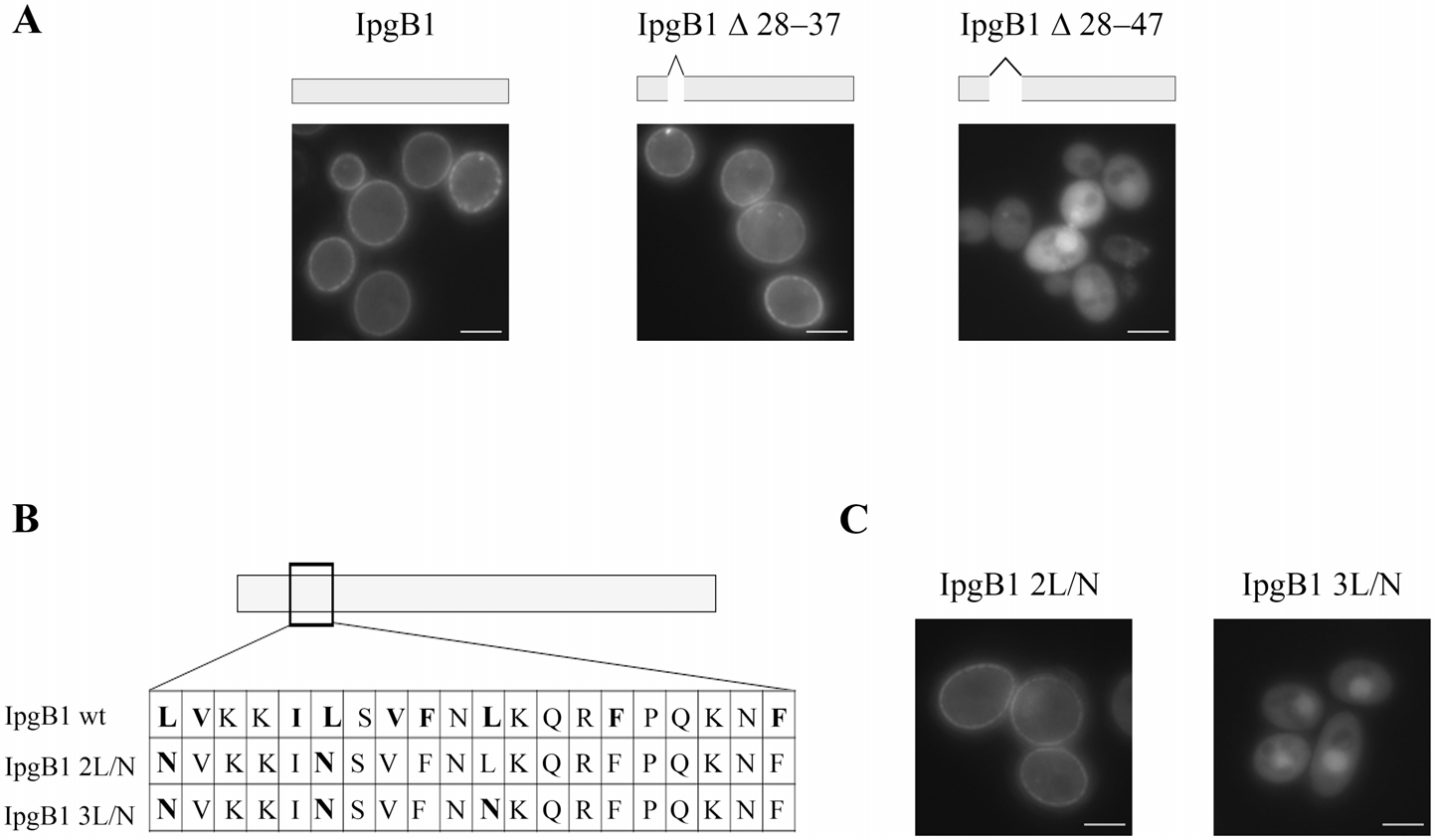
The chaperone binding domain of IpgB1 is located within its membrane localization domain. (A, C) Yeast expressing the designated IpgB1 alleles were visualized by fluorescence microscopy 4 h post-induction of protein expression. The images shown are representative of at least 3 independent experiments. All of the GFP positive yeast cells visualized for each condition exhibited the subcellular localization pattern shown. (B) Schematic representation of the IpgB1 MLD sequence. The hydrophobic leucines that were replaced by asparagines are indicated in bold.

We next investigated whether the ten residues present within the chaperone binding domain of IpgB1 are necessary to direct its membrane localization. As shown in Figure 3A, deletion of these 10 residues from intact wild type IpgB1 (IpgB1_Δ28–37) does not abrogate IpgB1 membrane localization. However, extension of the region to include 10 amino acids downstream of its CBD, residues 38–47 (IpgB1_Δ28–47) results in an allele that like free GFP (Figure 1B) localizes diffusely in yeast cells. Thus, we have identified a region of 20 amino acids required for the membrane localization of IpgB1.

At least three general mechanisms have been described to mediate the targeting of bacterial effectors to host cell membranes (for review, see [20]). First, numerous membrane localized effectors, including *Salmonella* SifA, another member of the WxxxE family of effectors, undergo lipidation modifications post-delivery into host cells that promote membrane targeting, i.e., palmitoylation, prenylation, and myristoylation [21], [22]. Second, at least in the case of *Salmonella*, coiled-coiled domains of multiple effectors assist in membrane targeting [23]. Lastly, multiple effectors encode membrane localization domains or MLDS, regions hypothesized to directly or indirectly promote interactions between effectors and membranes. MLDs are currently characterized as regions of effectors enriched in hydrophobic and charged residues that when deleted result in loss of effector membrane targeting [20], [24]–[27]. No specific sequence is shared.

Bioinformatic based analyses suggest that the residues required for IpgB1 membrane localization do not undergo any post-translational lipidation modifications nor encode a coiled-coil domain. However, similar to the regions of those effectors that encode MLDs, the region of IpgB1 required for membrane localization is enriched for hydrophobic as well as charged amino acids (Figure 3B). MLDs of variable lengths ranging from 20 to 90 residues have been mapped and the deletion of each of these regions, like residues 28–47 or IpgB1, results in loss of effector membrane targeting [20], [24]–[26].

Interestingly, the MLDs of *Yersinia* YopE, *Pseudomonas* ExoS and *Pseudomonas* ExoT share a conserved 22 amino acid symmetrical leucine-rich motif. In the case of ExoS substitution of the conserved hydrophobic leucines with polar asparagines disrupts its membrane localization [26]. While the length of the region of IpgB1 needed for membrane targeting is similar to ExoS, ExoT and YopE, the spacing of the leucines in the membrane targeting region of IpgB1 differs. Nevertheless, to test whether hydrophobic interactions also likely play a role in IpgB1 membrane targeting, we converted each of the leucines residues present within IpgB1 between amino acid 28 and 47 to asparagines. As observed with ExoS, when all three leucines are altered, IpgB1 no longer exhibits a membrane localization pattern (Figure 3C). Notably, all three leucines needed to be mutated, as the conversion of only leucines 28 and 33 to asparagines does not impair IpgB1membrane localization pattern of IpgB1 (Figure 3C). Therefore, comparable to ExoS, ExoT and YopE, the hydrophobic residues of the IpgB1 likely drive effector membrane targeting. Thus, we propose that this 20 amino acid region of IpgB1 also defines an MLD.

### Spa15 Promotes IpgB1 Stability by Masking Hydrophobic Residues within the MLD

Similar to IpgB1, the membrane localization domains of *Yersinia* YopO (YpkA), YopT and YopE overlap with their chaperone binding domains [27], [28]. Given the high prevalence of hydrophobic residues within MLDs and their propensity to be positioned within chaperone binding domains, Letzelter and colleagues proposed that the major role of chaperones is to mask hydrophobic MLDs to ensure that effectors remain soluble and stable within bacteria prior to their delivery via the secretion apparatus into host cells. Consistent with this hypothesis, YopO, YopT and YopE are unstable when heterologously expressed in *E. coli* in the absence of their cognate chaperones [28]. Furthermore, deletion of the hydrophobic MLD of YopO results in a stably expressed allele [28]. We next investigated whether this was the case for IpgB1 and its cognate chaperone, Spa15.

All of the effectors previously identified to contain a MLD bind class IA chaperones that are dedicated solely to their delivery into host cells. In contrast, IpgB1 is one of nine effectors that bind Spa15, a class IB chaperone [3], [7], [10]. Each of these nine Spa15-dependent effectors (OspB, OspC1, OspC2, OspC3, OspD1, OspD2, IpaA, IpgB1 and IpgB2) encodes a variant of the conserved chaperone binding domain (CCBD) sequence within its first 50 amino acids. Interestingly although residues within the CCBD sequence of IpgB1 are important for its membrane localization, none of the other eight Spa15-dependent effectors exhibits a membrane localization pattern when expressed in yeast [10], [11].

**Figure 4:**
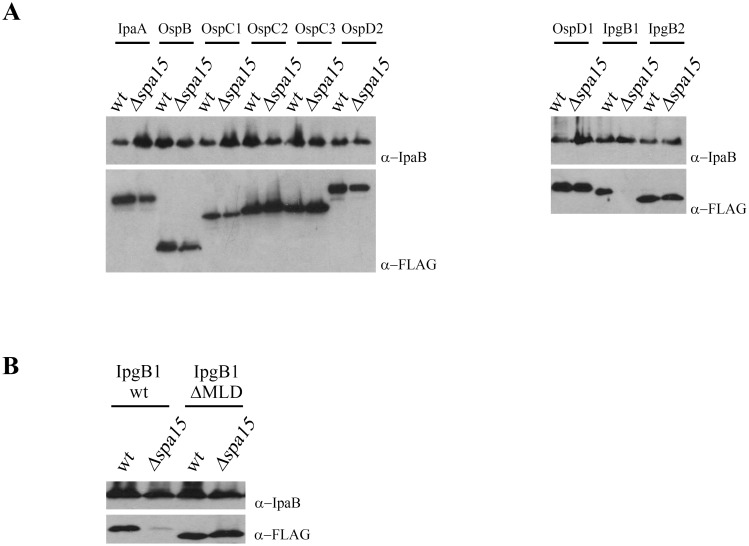
Spa15 masks the MLD of IpgB1 to promote effector stability. (A, B) Wild type and Δ*spa15 S. flexneri* strains that conditionally express the designated FLAG epitope-tagged alleles of each of the 9 Spa15-dependent effectors were grown at 37°C to an OD_600_ = 0.4 to induce expression of the *Shigella* T3SS. Subsequently, 30 minutes post-induction of the FLAG-tagged effectors, an equal amount of bacteria expressing each protein were pelleted and lysed. Protein lysates were separated by SDS-PAGE and immunoblotted with anti-FLAG and anti-IpaB antibodies. The latter serves as a control for loading and for induction of expression of the type 3 secretion apparatus. The blots shown are representative of at least 3 independent experiments.

Previous studies have reported that in *Shigella* the stability of IpgB1, but not IpgB2, is dependent on Spa15 [3], suggesting that Spa15 may not generally act to maintain effector stability. To investigate this possibility, we compared the relative amount of each of the nine Spa15-dependent effectors when expressed in wild type and *Δspa15 Shigella.* As shown in Figure 4A, steady-state levels of only IpgB1 is dependent on Spa15. As observed with YopO, deletion of the MLD of IpgB1 (IpgB1ΔMLD) restores protein stability even in the absence of Spa15 (Figure 4B). Thus, while Spa15 promotes IpgB1 stability by masking its hydrophobic MLD, the eight other Spa15-depenent effectors do not require Spa15 to maintain stability. These observations suggest that while a major function of dedicated class IA chaperones may be to promote stability of MLD-containing effectors, this is not the case for class IB chaperones like Spa15.

### The CBD Sequence of Spa15-dependent Effectors Is Necessary to Define these Proteins as Type 3 Secreted Substrates

**Figure 5:**
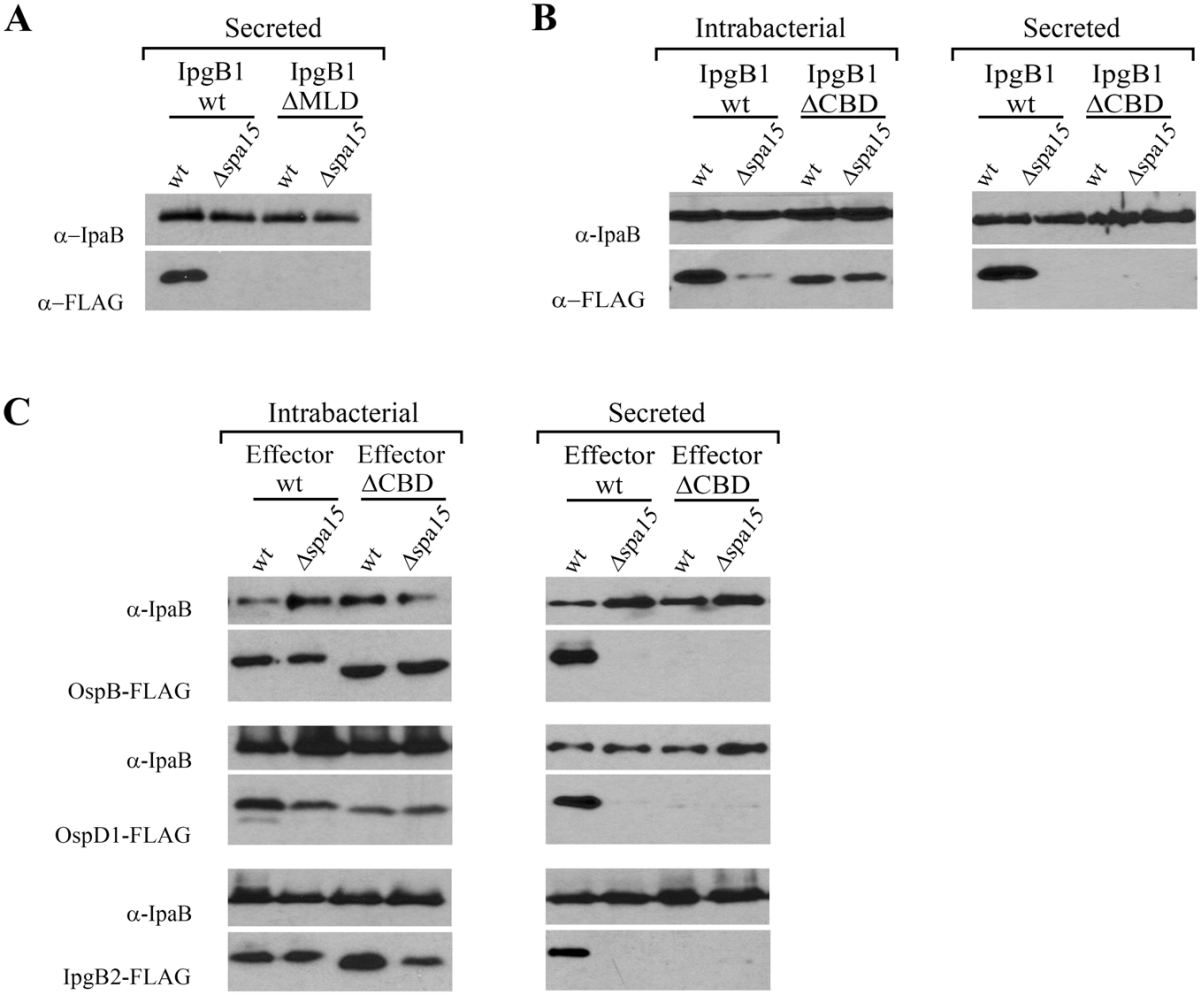
The CBDs of Spa15-dependent effectors are essential for their recognition as secreted substrates. (A, B, C) To assess levels of secreted effectors, wild type and Δ*spa15 Shigella* strains that conditionally express epitope-tagged alleles of the designated effectors were grown at 37°C to induce expression of the type 3 secretion apparatus. Thirty minutes post-addition of Congo red to induce type 3 secretion, culture supernatant proteins were TCA precipitated and separated by SDS/PAGE. (B, C) To assess intrabacterial levels of effectors, wild type and Δ*spa15 Shigella* strains that conditionally express the designated FLAG epitope-tagged alleles were grown at 37°C to an OD_600_ = 0.4. Subsequently, 30 minutes post-induction of expression of the designated FLAG-tagged effectors, equal numbers of bacteria expressing each effector were pelleted and lysed. Protein lysates were separated by SDS-PAGE. To determine levels of secreted and intrabacterial effectors, the SDS/PAGE gels were immunoblotted with anti-FLAG and anti-IpaB antibodies. Anti-FLAG antibody was used to assess effector secretion while anti-IpaB antibody was used as a control for loading and for induction of expression of the type 3 secretion apparatus. The blots shown are representative of at least 3 independent experiments.

Type 3 secretion chaperones have long been recognized to promote the delivery of effectors into host cells. However, in 2006, Letzelter and colleagues demonstrated that, at least for *Yesinia*, YopO deletion of the MLD results in a stable effector that is secreted in a chaperone-independent manner. This suggested that, in the context of MLD-containing effectors, chaperones act to promote effector stability but do not play a role in the recognition of the effector as a type three substrate [28]. We next investigated whether this is generally true and tested whether this is the case for IpgB1. Notably, as shown in Figure 5A, although IpgB1_ΔMLD is stable in both wild type and *Δspa15 Shigella* (Figure 4B), IpgB1ΔMLD is not recognized as a type 3 secreted substrate. Thus, at least for IpgB1 interactions with its cognate chaperone are required both to mask its hydrophobic MLD to promote stability and for its recognition as a T3SS secreted substrate.

Given that the CBD of IpgB1 is embedded within its MLD, we hypothesized that IpgB1ΔMLD is not secreted due to the absence of the CBD. Indeed, as shown in Figure 5B, while deletion of the ten residues of the CBD is sufficient to partially restore IpgB1 stability in the absence of Spa15, this allele, like IpgB1ΔMLD, is not recognized as a secreted substrate either in the presence or absence of Spa15. Similarly, we observe that deletion of the CBD of additional three Spa15-dependent effectors, IpgB2, OspB and OspD1, also results in stable alleles that are not recognized as type 3 secreted substrates (Figure 5C). With the exception of the deleted 10 residues that define each of their CBDs, the sequence of all four effectors is unaltered, including the N-terminal residues that encode their type 3 secretion signal sequences. Thus, in the case of *Shigella* class IB Spa15-dependent effectors, the N-terminal secretion signal sequence seems to not be sufficient to define these proteins as secreted substrates, rather the CBD or the chaperone-effector complex appears to play a major role in the recognition of Spa15 dependent effectors by the *Shigella* type 3 secretion apparatus.

## Discussion

Type 3 secreted effectors are multi-domain proteins optimized not only to be recognized as bacterial secreted substrates, but also to act within eukaryotic host cells. Generally, the N-terminal residues of effectors are devoted to protein delivery, while the downstream residues encode domains that target eukaryotic host cell proteins. However, there are increasing examples of how these two regions can overlap. For example, here we demonstrate that a single region of IpgB1 is essential both for its secretion from bacteria as well as its targeting to eukaryotic plasma membranes.

IpgB1 is one of ∼30 *Shigella* secreted effectors that are delivered into host cells during the course of an infection. Notably, of the 22 who have been studied as N-terminal GFP fusion proteins in yeast, IpgB1 is the only one that exhibits a membrane localization pattern [10], [11]. Interestingly, IpgB1 is a RhoGEF involved in the formation of the membrane ruffles that engulf and mediate uptake of these intracellular pathogens into host cells [3]–[6]. Thus, the efficient recruitment of IpgB1 to host cell membranes after the bacteria dock onto host cells presumably plays an important role in mediating invasion of *Shigella* into normally non-phagocytic intestinal epithelial cells [6].

Here, using the yeast model system, we have identified a region of twenty residues of IpgB1 that defines a membrane localization domain (MLD). Multiple effectors have now been identified to encode MLDs. While the domains of three, *Pseudomonas* ExoS, ExoT and *Yersinia* YopE, share a high degree of homology, MLDs can vary in terms of length and spacing of hydrophobic residues (for review see [20]). Interestingly, the MLDs of ExoS, ExoT and YopE are similar in length to that of IpgB1. However, while the hydrophobic residues of their MLDs are spaced such that they theoretically lie on the same face of an α-helix [29], this is not the case for IpgB1. It is unclear whether the MLDs directly bind membranes or membrane associated proteins. However, in the case of IpgB1, if the MLD promotes targeting by directly binding to a host cell protein, this protein or a close homology would need to be a conserved from yeast to mammals.

IpgB1 is one of nine *Shigella* effectors that bind Spa15, a class IB chaperone. Within their N-terminal 50 residues, each of these effectors encodes a variant of the conserved chaperone binding domain (CCBD) sequence [11]. The most highly conserved residues in this consensus sequence [11], those at positions 1, 4 and 7, are hydrophobic in character, yet IpgB1 is the only one of the nine effectors that exhibits a membrane localization pattern when expressed in yeast. Notably, a comparison of the overall character of the residues of the nine Spa15-dependent effectors that encompass the CCBD plus the downstream ten residues, the region required for the membrane localization of IpgB1, demonstrates that the overall hydrophobicity of IpgB1 is greater than any other effector, again supporting the hypothesis that this region of IpgB1 drives membrane targeting.

The overlapping of the membrane localization and chaperone binding domains of IpgB1 and multiple other effectors is perhaps not that surprising. First, as exemplified by the conserved residues of the CCBD sequence and observed in chaperone-effector co-crystal structures, hydrophobic interactions play major roles in mediating chaperone-effector binding interactions [30], [31]. Furthermore, for multiple MLD-containing effectors, including IpgB1, cognate chaperones promote effector stability within bacteria by masking the hydrophobic residues. These observations led Letzelter and colleagues to propose that a major role of chaperones, at least for MLD-containing effectors, is to promote effector stability within bacteria rather than the targeting of the chaperone-effector complex to the secretion apparatus [28]. While this may prove to be the case for class IA chaperones that mediate the play a role in the secretion of only 1–2 effectors, this does not appear to be the case with Spa15, a class IB chaperone, which mediates the secretion of multiple effectors, including IpgB1 into host cells. Rather our data supports the hypothesis that the chaperone-effector complex plays a major role in identifying proteins as secreted substrates [30], [32], given that deletion of the ten residues corresponding to the CCBD sequence of at least four Spa15-dependent effectors results in loss of their secretion despite the presence of an intact N-terminal secretion sequence.

Lastly, it is interesting to speculate that Spa15 might have originally been devoted to the delivery of IpgB1 into host cells. Spa15 is a class IB type 3 secretion chaperone. In contrast to class IA chaperones that are encoded within operons alongside their cognate 1–2 effectors, the gene encoding Spa15 is located adjacent to those for components of the secretion apparatus. Interestingly, genes for only two effectors that bind Spa15, IpgB1 and IpaA, are encoded in close proximity to that which encodes Spa15. And, of these two, there is evidence that IpaA is secreted via both chaperone-dependent and chaperone-independent pathways [7]. Furthermore, IpgB1 is the only one of the 9 Spa15-dependent effectors that doesn’t directly interact with class IB chaperones from related bacteria that also bind to CCBD sequence containing effectors [11]. Thus, given that chaperones can play a role in determining the hierarchy of delivery of effectors into host cells, if Spa15 binds with highest affinity to IpgB1 perhaps this interaction ensures that this effector, which plays a major role in host cell invasion, is one of the first to be delivery into host cells.

## Supporting Information

Table S1 **List of oligonucleotides used to generate fragments via PCR for cloning into Gateway entry vectors.**
(DOC)
