# Supplementary material for: A Multifunctional Region of the Shigella Type 3 Effector IpgB1 Is Important for Secretion from Bacteria and Membrane Targeting in Eukaryotic Cells
Source: PLoS One. 2014 Apr 9;9(4):e93461. doi: 10.1371/journal.pone.0093461 (PMC3981709; doi:10.1371/journal.pone.0093461)
Supplement: Table S1 — List of oligonucleotides used to generate fragments via PCR for cloning into Gateway entry vectors. (DOC) [file pone.0093461.s001.doc]

| Table S1: | List of oligonucleotides used to generate fragments via PCR for cloning into Gateway entry vectors |
| --- | --- |
| Gene | Primers |
|  |  |
| Universal 5’ | GGGGACAACTTTGTACAAAAAAGTTGGCGAAGGAGATAGAACCATG |
| IpgB1 Wt | 5’ CGAAGGAGATAGAACCATGCAAATTCTAAACAAAATACTTC |
|  | 3’ GGGGACAACTTTGTACAAGAAAGTTGGTTAATTTGTATTGCTTTGACGGTAT |
| IpgB1 Nterminal | 5’ TCATCAACCAAAATAAATACTAGTATTTTGAGCTCTGGATGCCCGGTGAATATAAATAAA |
|  | 3’CAATCTGTAATATTTTCACCGATTTGTTCTGAAACAAAATTCTTCTGAGGAAAGCGTTGTTTT |
| IpgB1 Chimera A | 5’ TCATCAACCAAAATAAATACTAGTATTTTGAGCTCTGGATGCCCGGTGAATATAAATAAA |
|  | 3’GCTGTCCCTGATTTTATTTATATTCACCGGGCATCCAGAGCTCAAAATACTAGTATTTATTTTG |
| IpgB1 Chimera C | 5’ AGTCGCAATAAGATGGGGSAAAAGGATAGTAAAAATATCATCATTAAAACAACGCTTT |
|  | 3’ AAAGCGTTGTTTTAATGATGATATTTTTACTATCCTTTTCCCCATCTTATTGCGACT |
| IpgB1 2L/N | 5’ TCGTTAAGTCGCAATAAGAATGTAAAGAAAATAAATTCTGTTTTTAACTTA |
|  | 3’TAAGTTAAAAACAGAATTTATTTTCTTTACATTCTTATTGCGACTTAACGA |
| IpgB1 3L/N | 5’ AGTCGCAATAAGGCTGTAAAGAAAATAGCATCTGTTTTTAACGCAAAACAACGC |
|  | 3’ GCGTTGTTTTGCGTTAAAAACAGATGCTATTTTCTTTACAGCCTTATTGCGACT |
| IpgB1Δ28-37 (CCBD) | 5’ ATTTGATTCGTTAAGTCGCAATAAGTTAAAACAACGCTTTCCTCAGAAGA |
|  | 3’ TCTTCTGAGGAAAGCGTTGTTTTAACTTATTGCGACTTAACGAATCAAAT |
| IpgB1Δ28-47 | 5’ TTTGATTCGTTAAGTCGCAATAAGGGATGCCCGGTGAATATAAATAAA |
|  | 3’ TTTATTTATATTCACCGGGCATCCCTTATTGCGACTTAACGAATCAAA |
| IpgB1 E80A | 5 CAACTATTTTGTTGGATGAGCCAAGCGCGAACCACTTATGTCTCTTCA |
|  | 3 TGAAGAGACATAAGTGGTTCGCGCTTGGCTCATCCAACAAAATAGTTG |
| IpgB2 wt | 5’ CGAAGGAGATAGAACCATGCTTGGAACATCTTTTAATAATT |
|  | 3’ GGGGACAACTTTGTACAAGAAAGTTGGTCAGAAAGGCGATTCTAAATTTGTA |
| IpgB2 W62A | 5’TCAGAACAAATCGGTGAAAATATTACAGATGCGAAAAATGATGAAAAAAAAGTC |
|  | 3’GGATACGTAGACTTTTTTTTCATCATTTTTCGCATCTGTAATATTTTCACCGAT |
| IpgB2 Nterminal | 5’ TTTAACTTAAAACAACGCTTTCCTCAGAAGAATTTTGTTTCAGAACAAATCGGTGAAAAT |
|  | 3’ GCTGTCCCTGATTTTATTTATATTCACCGGGCATCCAGAGCTCAAAATACTAGTATTTAT |
| IpgB2 Chimera B | 5’ TTTAACTTAAAACAACGCTTTCCTCAGAAGAATTTTGTTTCAGAACAAATCGGTGAAAAT |
|  | 3’CCAATCTGTAATATTTTCACCGATTTGTTCTGAAACAAAATTCTTCTGAGGAAAGCGTTGTTTT |
| IpgB2 Chimera D | 5’ AAGGTGGATGAGATTATCCGCTGTACACTTGTAAAGAAAATATTATCTGTTTTTAAC |
|  | 3’ AGAGCTCAAAATACTAGTATTTATTTTGGTGTTAAAAACAGATAATATTTTCTTTACAAG |
| IpgB2ΔCCBD | 5’ GGAAAGGTGGATGAGATTATCCGCTGTACAACCAAAATAAATACTAGTATTTTGAGCTCT |
|  | 3’ AGAGCTCAAAATACTAGTATTTATTTTGGTTGTACAGCGGATAATCTCATCCACCTTTCC |
| OspD1ΔCCBD | 5’ GGCAGCAATACTGCTAATGAAAATAAAGGAACCGCTATATCCCACGCCATCAATGAA­­GAA |
|  | 3’ TTCTTCATTGATGGCGTGGGATATAGCGGTTCCTTTATTTTCATTAGCAGTATTGCTGCC |
| OspBΔCCBD | 5' AAAAAGAATGAAAGCATATCAGATATTGCATCATCATGTACTCACCCAAAAGCAGCATTG |
|  | 3’ CAATGCTGCTTTTGGGTGAGTACATGATGCAATATCTGATATGCTTTCATTCTTTT |
| GFP-MLD | 5’CGAAGGAGATAGAACCATGAGTAAAGGAGAAGAACTTTTCA |
|  | RoundB’AAACAGATAATATTTTCTTTACAAGTTTGTATAGTTCATCCATGCCATGT |
|  | RoundC’AGGAAAGCGTTGTTTTAAGTTAAAAACAGATAATATTTTCTTTACA |
|  | 3’GGGGACAACTTTGTACAAGAAAGTTGGTAATTCTTCTGAGGAAAGCGTTGTTTT |
